# Spectral diversity of photosystem I from flowering plants

**DOI:** 10.1007/s11120-022-00971-2

**Published:** 2022-10-19

**Authors:** Peter R. Bos, Christo Schiphorst, Ian Kercher, Sieka Buis, Djanick de Jong, Igor Vunderink, Emilie Wientjes

**Affiliations:** Laboratory of Biophysics, Wageningen University, P.O. Box 8128, 6700 ET Wageningen, The Netherlands

**Keywords:** Light harvesting, Photosystem I, Fluorescence, Absorption, Spectroscopy

## Abstract

Photosystem I and II (PSI and PSII) work together to convert solar energy into chemical energy. Whilst a lot of research has been done to unravel variability of PSII fluorescence in response to biotic and abiotic factors, the contribution of PSI to in vivo fluorescence measurements has often been neglected or considered to be constant. Furthermore, little is known about how the absorption and emission properties of PSI from different plant species differ. In this study, we have isolated PSI from five plant species and compared their characteristics using a combination of optical and biochemical techniques. Differences have been identified in the fluorescence emission spectra and at the protein level, whereas the absorption spectra were virtually the same in all cases. In addition, the emission spectrum of PSI depends on temperature over a physiologically relevant range from 280 to 298 K. Combined, our data show a critical comparison of the absorption and emission properties of PSI from various plant species.

**Supplementary Information:**

The online version contains supplementary material available at 10.1007/s11120-022-00971-2.

## Introduction

Photosynthesis is driven by light absorbed by photosystem I (PSI) and photosystem II (PSII). Both photosystems are located in the thylakoid membrane of oxygenic photosynthetic organisms (Blankenship 2021). The supramolecular PSI complex oxidises plastocyanin and photoreduces ferredoxins in the photosynthetic electron transport chain (Gobets and van Grondelle 2001). In plants PSI is composed of multiple proteins that can be divided in two moieties: (i) the core, harbouring the reaction centre, all electron transport cofactors, 102 chlorophyll (Chl) *a* molecules and ~ 22 β-carotenes and (ii) an outer light-harvesting complex (LHCI) composed of four gene products (Lhca1-4), which coordinate Chl *a*, Chl *b*, lutein, violaxanthin and β-carotene (Ben-Shem et al. 2003; Amunts et al. 2007; Wientjes and Croce 2011). The PSI core is much conserved over the course of evolution and can be traced back to cyanobacteria, the oldest known clade of oxygenic photosynthetic organisms (Amunts and Nelson 2008; Cardona 2018, Sánchez‐Baracaldo and Cardona 2020). Due to the low mutation rate of PSI core proteins the structure and pigment organisation of the PSI core from plants, algae and cyanobacteria is almost identical (Jordan et al. 2001; Galka et al. 2012; Qin et al. 2015; Mazor et al. 2017; Pan et al. 2018; Steinbeck et al. 2018).

Contrarily, LHCI has emerged later in evolution in green algae and higher plants and is found to be more variable between species than the PSI core complex (Green 2003; Croce and van Amerongen 2020; Pan et al. 2020). LHCI of *Chlamydomonas reinhardtii* consists of nine gene products (Lhca1-9) that form two parallel concentric half rings, whilst the LHCI complex of higher plants only forms a single half-moon shaped belt around PSI, organised as an Lhca1/4 and Lhca2/3 dimer (Amunts et al. 2007; Drop et al. 2011; Wientjes and Croce 2011; Su et al. 2019; Suga et al. 2019). In plants a fifth protein Lhca5 can be present in low concentrations and can substitute for Lhca4 (Klimmek et al. 2006; Wientjes et al. 2009). Recently it was reported that the optical properties of LHCI from different higher plant species can differ (Chukhutsina et al. 2020). A distinct feature of LHCI of higher plants is the absorption of photons with a wavelength > 700 nm by Chls which are called red forms. These red forms slow down excitation energy trapping but increase the range of wavelengths plants can use for photosynthesis (Croce et al. 2000; Le Quiniou et al. 2015). Red forms can arise when two Chls with transition dipole moments that are parallel and in line have strong electronic interaction (Van Amerongen and Van Grondelle 2000). The effect can become stronger when the Chls experience different polar environments, leading to the mixing of excitonic states with charge transfer states (Gobets et al. 1994; Romero et al. 2009; Novoderezhkin et al. 2016). Lhca3 and Lhca4 harbour such coupled Chls and therefore give rise to the most red-shifted absorption and emission bands of PSI (Morosinotto et al. 2003; Croce et al. 2007). The organisation of these low-energy red forms in Lhca3 and Lhca4 is very similar (Wientjes et al. 2011a, b). Excitations residing on the red-shifted Chls must overcome their energy deficit with environmental heat for transfer to neighbouring Chls. Decreasing the temperature leads to an increase of the time the excitation resides on the red forms and, as such, the fluorescence of the low-energy Chls becomes more profound. This has been demonstrated over the temperature range from 17 to 280 K (Croce et al. 1998; Jelezko et al. 2000). The effect of temperature, on the PSI emission spectrum and intensity, in the biological relevant range has so far not been investigated.

Regardless of slower trapping due to red-shifted Chls, PSI is arguably the most efficient nanomachine in nature, using almost every absorbed photon for charge separation (Amunts and Nelson 2008). Since PSII has a higher fluorescence yield than PSI, most leaf Chl fluorescence is emitted from Chls affiliated with PSII. PSII emission has been well studied and is known to vary due to differences in light conditions and plant stresses (Dau 1994; Goltsev et al. 2016). Contrastingly, less is known about the fluorescence emission properties of PSI. First of all, because its weak emission makes it difficult to obtain samples which are pure enough to study the properties of PSI and not that of PSII contaminations. Furthermore, the emission intensity of PSI is very similar for open and closed reaction centres and not affected by non-photochemical quenching processes (Itoh and Sugiura 2004; Wientjes and Croce 2012; Porcar-Castell et al. 2014). However, PSI fluorescence can contribute significantly to leaf fluorescence depending on the detection wavelength and the level of (non)-photochemical quenching of PSII emission. At 680 nm, the fluorescence signal is dominated by PSII, but with detection above 720 nm, the PSI contribution can reach up to 40% of the fluorescence signal from whole leaves (Agati et al. 2000; Franck et al. 2002). Therefore, knowing the emission spectra of both photosystems is essential for a correct interpretation of in vivo fluorescence data. Especially pulse amplitude modulation (PAM) measurements and remote sensing applications, techniques that commonly use detection wavelengths of in vivo fluorescence > 700 nm, would benefit from an accurate correction with a PSI fluorescence spectrum (Porcar-Castell et al. 2014).

The PSI trapping kinetics have been studied with time-resolved fluorescence measurements before (Mukerji and Sauer 1993; Pålsson et al. 1995; Croce et al. 2000; Ihalainen et al. 2002, 2005; Van Oort et al. 2008; Wientjes et al. 2011a, b; Jennings et al. 2013; Akhtar and Lambrev 2020). However, a wide range of average trapping times ranging from 40 to 64 ps at room temperature or even 99 ps at 280 K have been reported. This difference might be due to variations in the plant species that were investigated, but could also be due to isolation methods or measurement techniques. Recently it has been shown that the PSI emission spectrum and trapping kinetics varies from species to species (Chukhutsina et al. 2020). These results indicate that the PSI spectrum is not as invariable as thought. To disentangle the contribution of PSI and PSII of total leaf fluorescence we need to have accurate knowledge of the PSI spectrum, how it is affected by temperature and how it potentially differs between plant species.

Here, we used an array of biochemical and optical techniques to study the biological variability of PSI. To this end, we isolated PSI-LHCI supercomplexes from 5 different angiosperms (flowering plants), namely the sun-tolerant eudicot species *Arabidopsis thaliana* and *Spinacia oleracea* and the monocot species *Zea mays*, *Spathiphyllum wallisii* and *Calathea roseopicta* [in lesser used official nomenclature *Goeppertia roseopicta* (Borchsenius et al. 2012)]. The latter two are houseplants introduced from the South American tropical forests and are adapted a shade environment (Schott and Endlicher 1832; Van Huylenbroeck et al. 2018). *Zea mays* is also cultivated from South America, but is a sun-tolerant species (Benz 2001). The diversification of monocots and eudicots is hypothesised to have occurred around 130 million years ago which is therefore the maximum time between the most-recent common ancestor of two species in this study (Moore et al. 2007).

Since canopy shade light is enriched in far-red light (Rivadossi et al. 1999; Johnson and Wientjes 2020), plants that adapted to a niche in a shaded environment could benefit from using a broader spectrum of light, especially in the far-red part. Therefore, we hypothesise that the plants from the tropical rainforest, *S. wallisii* and *C. roseopicta* will have red-shifted red forms compared to the crop/pioneer species *A. thaliana*, *S. oleracea* and *Z. mays*. For sun-tolerant species the extended lifetime and the accompanied risk of damage to the photosystems could have suppressed adaptation to more red-shifted Chls. We found, based on multiple technical and biological replicas, significant differences in the emission properties of PSI of the five studied plant species. Moreover, we show that the PSI emission spectrum and intensity is affected by changes in temperature within a biologically relevant range (280–298 K).

## Materials and methods

### Plant material

*Arabidopsis thaliana* and *Zea mays* were grown in a plant cabinet at 60% humidity, 125 µmol/m/^2^/s, and 8 h day. Plants from both species were harvested after 6–8 weeks. *Spinacia oleracea* was purchased at the local supermarket. *Spathiphyllum wallisii* and *Goeppertia/Calathea roseopicta* were bought at the local gardening shop and grown in a living room without direct sunlight. The species were identified with the Pl@ntnet app (Identify, explore and share your observations of wild plants. Pl@ntNet. (n.d.). Retrieved January 25, 2022, from https://identify.plantnet.org/).

### Protein sequence alignment

The protein sequences of Lhca1-4 of *A. thaliana* were acquired from the Arabidopsis Information Resource (TAIR) (Berardini et al. 2015). Orthologs in *Z. mays* and *S. oleracea* were detected using the “plant orthologs” section at the TAIR webpage of the specified protein. In case of multiple orthologs per species, all were aligned to the *A. thaliana* protein sequence and the sequences with the highest degree of cover were chosen. A deep learning approach was used to detect and remove chloroplast transit peptides from the sequences (Armenteros et al. 2019). Chloroplast transit peptides were detected with high probability (> 0.77). Sequences were aligned with the web-based tool multiple sequences alignment Clustal Omega (Sievers et al. 2011; McWilliam et al. 2013) from which percent identity was obtained.

### PSI-LHCI isolation

Thylakoids were harvested according to a protocol adapted from Caffari et al. (Caffarri et al. 2009). In short, leaves were homogenised quickly in buffer 1 (400-mM sorbitol, 5-mM EDTA, 10-mM NaHCO_3_, 5-mM MgCl_2_, 20-mM tricine and 10-mM NaF) in a blender. The solution was filtered through a 400-µm and 100-µm mesh sized filter and centrifuged for 3 min at 2000 g. The pellet was carefully resuspended in buffer 2 (300-mM sorbitol, 5-mM MgCl_2_, 20-mM tricine, 2.5-mM EDTA, 10-mM NaF and 10-mM NaHCO_3_) and the chloroplasts were again pelleted for 3 min at 2000 g. The pellet was resuspended in buffer 3 (5-mM MgCl_2_, 2.5-mM EDTA, 10-mM NaF and 20-mM Hepes) and centrifuged for 10 min at 10,000 g. The centrifuge was cooled to 4 °C and the material was kept on ice as much as possible. The pellet was resuspended in buffer 1 and stored at − 80 °C until further use.

PSI-LHCI complexes were isolated according to a slightly modified protocol of Wientjes et al. from a second sucrose gradient (Wientjes et al. 2009). Solubilisation happened with 0.6% α-DM or β-DM and sucrose gradients were centrifuged for 16–20 h at 40.000 rpm in a Beckman SW-41Ti rotor (1.97 × 10^5^ g). The lowest green band contained PSI-LHCI and was isolated with a syringe, concentrated and loaded on a second sucrose gradient. The lowest band was isolated with a syringe, concentrated and stored at − 80 °C. The one but lowest band on the first sucrose gradient contained the PSII core and was isolated and stored at − 80 °C.

### Pigment analysis

Pigments were extracted in 80% acetone and absorption spectra were recorded from 350 to 750 nm. Spectra were fitted with the spectra of individual pigments as described by Chazaux et al. to determine Chl concentration and Chl a/b ratio (Chazaux et al. 2022).

### Gel electrophoresis

The SDS-PAGE electrophoresis protocol was adapted from Laemmli (1970). A ratio of 32:1 acrylamide/bisacrylamide was used to a concentration of 15% in the running gel and 5% in the stacking gel. 2 M urea was incorporated in the gel. Samples were diluted to loading concentration (~ 150 µg/mL Chl) in the presence of DTT and were heated to 70 °C for 10 min. Around 2 µg Chl was loaded per lane. The gel was stained with Coomassie R and imaged with a Bio-rad Universal Hood II.

### Spectroscopy

Samples were diluted to 10 µg/mL in buffer 1. Absorption spectra were recorded with a Cary-4000 UV–VIS spectrophotometer (Agilent Technologies, Inc., Santa Clara, USA) from 350 to 800 nm. Absorption at 750 nm was set to zero and spectra were corrected for scattering by subtracting a linear line with the slope set to the average slope of the spectrum between 750 and 800 nm. Spectra were normalised by the area between 650 and 750 nm. Technical replicates were averaged and significant differences between biological replicates were determined with a Tukey test. Biological replicates were averaged and plotted.

Fluorescence spectra in a 1-cm cuvette in a Fluorolog 3.22 spectrofluorometer (HORIBA Jobin Yvon, Longjumeau, France) with 435-nm excitation (10-nm slit width) and 600–800-nm emission were recorded. The temperature was regulated with a water bath and measured with a temperature sensor. The temperature was increased stepwise with 4–5 degrees steps from 280 to 298 K whilst cooling the sample in between to minimise damage. Damage was checked by taking a second spectrum at 280 K after the 298-K measurement. Samples were recorded three times with an integration time of 0.4 s and averaged. To quantify the change in the red-form emission, the minimal fluorescence value was set to zero and the data were normalised to the maximum value between 675 and 690 nm to correct for the damage to the photosystem that resulted in a lower fluorescence. The sum of intensity between 700 and 800 nm was calculated and the resulting value from the measurement at 293 K was set to 1. Where applicable, data were interpolated and values at 298 K and 286 K were averaged.

Steady-state 77-K fluorescence measurements were performed in a glass Pasteur pipette (pathlength ~ 1 mm) in a glass Dewar filled with liquid nitrogen with excitation wavelength 435 nm and emission recorded from 600 to 800 nm with a 1-nm step-size and an integration time of 0.4 s. Spectra were recorded three times and averaged.

Room temperature and 77-K spectra were normalised to the maximum fluorescence value > 700 nm, smoothed with the Savitzky–Golay filter, 20 points of window, 2nd polynomial order and normalised again using Origin (Origin, Version 2020b. OriginLab Corporation, Northampton, MA, USA). Where applicable, technical replicates were averaged and significance was determined by a Tukey test (*p* < 0.05) between different species. Averages were taken per species and plotted. Visualisation in boxplots was achieved with Origin. Temperature-controlled spectra between 280 and 298 K were also smoothed with the Savitzky–Golay filter, 20 points of window, and 2nd polynomial order with Origin.

### Time-resolved spectroscopy

Time-resolved fluorescence measurements were performed with a streak-camera setup as described earlier (Van Oort et al. 2009). Excitation wavelength was 400 nm and time window was set to 800 ps. Samples were diluted to 10 µg/mL Chl in a 1 × 1-cm cuvette and stirred continuously. Temperature of the sample was regulated with a Peltier-based thermostat connected to a copper sample holder and cooled with a water bath.

The collected images were corrected for background signal and spatial variations in detector sensitivity. Corrected images were averaged over 5 nm and globally analysed with the specialised software Glotaran to construct decay-associated spectra (DAS) (Mullen and Van Stokkum 2007; Snellenburg et al. 2012).

4 DAS fitted the PSI streak images best. Spectra were normalised to the total area under the graphs and interpolated using Origin (Origin, Version 2020b. OriginLab Corporation, Northampton, MA, USA). DAS1 described excitation energy transfer within PSI. DAS2 and DAS3 described the decay of PSI, whilst DAS4 described the decay of contaminations, like free Chls, LHCII or PSII. To calculate the average lifetime of PSI, DAS2 and DAS3 were used:

$\left\langle \tau \right\rangle = {\text{Area}}_{{{\text{DAS}}2}} \times \tau_{2} + {\text{Area}}_{{{\text{DAS}}3}} \times \tau_{3} /\left( {{\text{Area}}_{{{\text{DAS}}2}} + {\text{Area}}_{{{\text{DAS}}3}} } \right)$, with ${\text{Area}}_{{\text{DAS}}\#}$ the area under the DAS, and ${\tau }_{\#}$ the fluorescence lifetime associated with the DAS. The emission maximum of DAS3 was determined in the section between 703 and 778 nm. Values for lifetime and *λ*_max_ were averaged between technical replicates and statistical differences between biological replicates were determined with a one-way ANOVA and a Tukey test (*p* < 0.05) using Origin (Origin, Version 2020b. OriginLab Corporation, Northampton, MA, USA.).

### Calculation PSI contribution to total fluorescence

Fluorescence spectra at various temperatures of PSI from *S. oleracea* were used. For the PSII spectrum we recorded the emission of freshly prepared grana membranes prepared according to (Barbato et al. 2000). Since no temperature dependence of PSII was observed, this PSII spectrum was used for all temperatures. The spectra were normalised to the total area and multiplied with the transmission spectrum of the RG9 filter, acquired from Schott by linear interpolation between given transmission data points in the RG9 datasheet (Schott AG, Mainz, Germany). This filter is common in many PAM systems. Spectra were multiplied with their lifetime (69 ps for PSI as determined with time-resolved spectroscopy, 224 ps for open PSII reaction centres (Wientjes et al. 2013a, b) and 1.6 ns for closed PSII reaction centres (Roelofs et al. 1992; Matsubara and Chow 2004; Rizzo et al. 2014)) and added up to get the total fluorescence spectrum in F_0_ and F_M_ situation. Equal excitation of the two photosystems was assumed. The PSI contribution to the total fluorescence in F_0_ and F_M_ (PSI/(PSI + PSII)) and F_v_/F_M_ (F_v_/F_M_ = 1 − F_0_/F_M_) was calculated.

## Results

### Protein composition of PSI-LHCI isolates

**Fig. 1:**
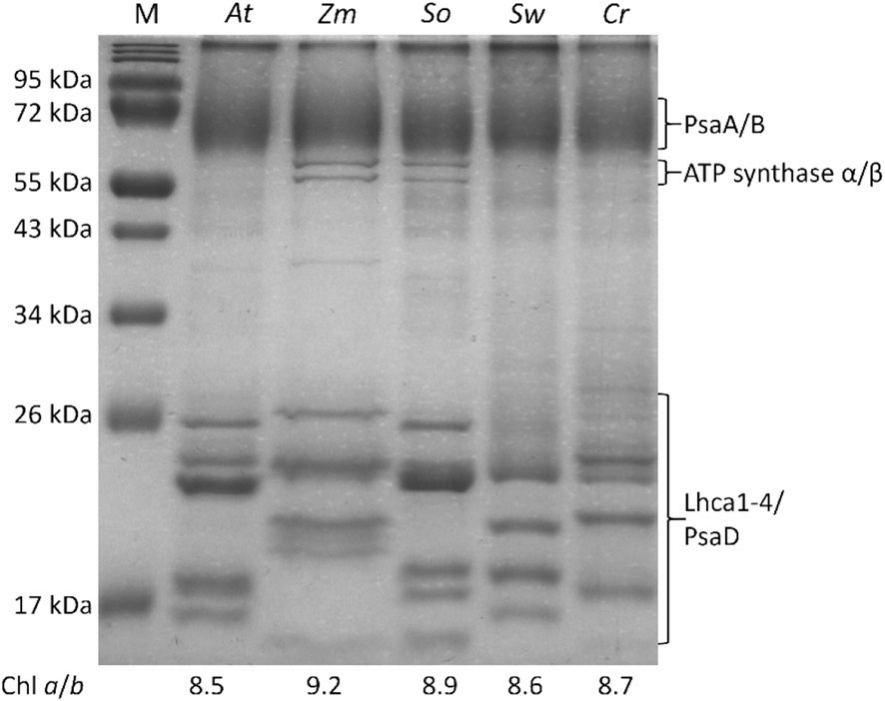
SDS-PAGE of PSI-LHCI complexes from *Arabidopsis thaliana* (At), *Zea mays* (Zm), *Spinacia oleracea* (So), *Spathiphyllum wallisii* (Sw) and *Calathea roseopicta* (Cr). Also a marker (M) is loaded and the bands annotated with their molecular weight. 2–2.5 µg Chl was loaded per lane. Chl* a*/*b* ratios of the loaded samples are listed below the lanes

PSI-LHCI complexes from five plant species were isolated and purified on sucrose gradients and subjected to an array of spectroscopic and biochemical techniques. Firstly, the protein content of the isolates was examined on an SDS-PAGE gel to identify the composition of PSI-LHCI in the different species and potential contaminations (Fig. 1). In all lanes, the core-subunits of PSI, PsaA and PsaB, are clearly visible. Two smaller bands around 60 kDa can be seen in *Z. mays* and *S. oleracea*, which are most likely originating from ATP synthase α and β subunit. Since these proteins do not contain pigments, they do not interfere with the spectroscopic measurements (Tian et al. 2017). In the lane of *A. thaliana,* five bands between 26 and 17 kDa are visible, which correspond to Lhca1-4 and PsaD (Ballottari et al. 2007). The migration behaviour of the Lhca bands of *S. oleracea* is very similar to the ones from *A. thaliana*, but the bands of the other three species differ. The large variation in migration behaviour of the Lhcas from the investigated species indicates that there is a variation in the lengths and/or amino acid composition of the polypeptide. This variation is less apparent in the Chl *a*/*b* ratios of the samples loaded on this gel. In addition, a high degree of similarity is observed in the protein sequences of *Z. mays*, *A. thaliana*, and *S. oleracea*, the three species of which the DNA sequences are known (90–94% between *A. thaliana* and *S. oleracea* and 85–89% between *A. thaliana* and *Z. mays*, see Supplementary Table S1). Despite the high similarity, repeated SDS-PAGE of different PSI-LHCI isolates show consistently a different migration behaviour of Lhca proteins between the species. The sizes of the mature proteins of these three species differ maximally 0.3 kDa. Therefore, the differences in migration behaviour must be due to the variation in amino acid composition. Indeed, small variations in the amino acid composition of membrane proteins can have large effects on the SDS-PAGE migration behaviour (Rath et al. 2009).

### PSI fluorescence varies between species

**Fig. 2:**
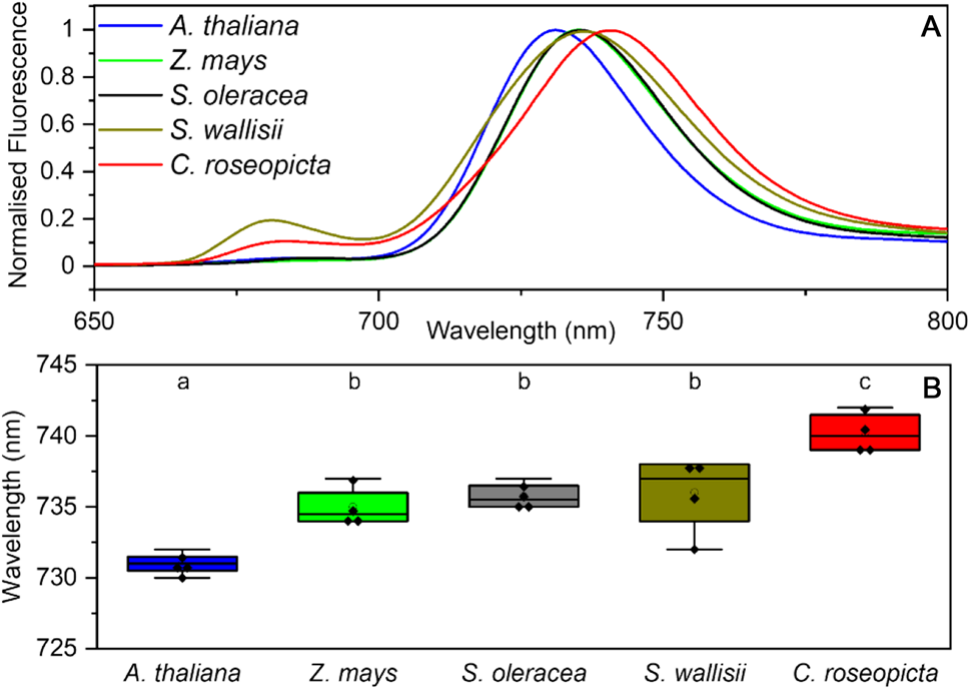
77-K fluorescence measurements on PSI from five plant species. **A** Average PSI spectra recorded at 77 K normalised to the emission maximum. Excitation at 435 nm. **B** Boxplot of the emission maxima. The boxes express 25th and 75th percentile, with median (line) and mean (open circle) indicated. Whiskers indicate the 5th and 95th percentile. Significant groups as determined by a Tukey test are indicated with letters (*p* < 0.05). Number of biological replicates is 4 (*N* = 4) for all species

At 77 K, fluorescence of the red forms of PSI is very profound, since the uphill energy transfer from a red-shifted Chl to the bulk Chls is severely slowed down due to the lack of environmental heat. Less environmental heat means a higher chance of the excitation residing on one of the low-energy Chls and eventually emitting a photon. Steady-state-fluorescence measurements on PSI complexes of different plant species at 77 K allow us to chart their difference in fluorescence spectra and especially in the red-emitting Chls. Moreover, the integrity of the complexes and contaminations can be examined at this temperature as mainly PSI emits around 730–740 nm, whilst PSII and free Chls *a* emit predominantly in the 670–690-nm region. *S. wallisii* and to a lesser extent *C. roseopicta* are the only two isolates with notable contamination with non-PSI bound pigments, as can be seen by a second fluorescence peak around 680 nm (Fig. 2 and Supplementary Fig. S1). Most likely these stem from PSII complexes or free Chls. Since the contamination in *S. wallisii* could not be diminished with a second sucrose gradient and increased substantially after being subjected to temperature changes, this could also point towards instability of the PSI complex for this plant species.

The 77-K emission spectra of the PSI complexes were compared with the emission of the intact thylakoid membranes to check if PSI isolation affects its emission properties (Supplementary Fig. S2). Perfect overlap, signifying no effect of the isolation, was observed for *A. thaliana, Z. mays*, and S. *oleracea.* Instead, the spectra of isolated PSI from *S. wallisii* and *C. roseopicta* show a broader and a few nm blue-shifted spectrum when compared to the intact thylakoids. This suggests that these PSI complexes are indeed less stable in detergent.

Variations in the PSI fluorescence spectra at < 700 nm can be explained by contamination with other compounds, but differences at wavelengths > 700 nm are likely due to diversity in PSI across species. In Fig. 2 it can be observed that the emission maximum of PSI from *A. thaliana* (*λ*_max_ = 731 ± 0.4 nm) is blue shifted and the spectrum of *C. roseopicta* (*λ*_max_ = 740 ± 0.8 nm) red shifted with regard to the complexes of the other species. Significant differences are found when comparing the *λ*_max_ of the different PSI complexes (Fig. 2). It is interesting to note that *C. roseopicta* showed an even more red-shifted emission maximum in intact thylakoids, with an emission maximum of 745 nm (Supplementary Fig. S2). The emission maxima of *Z. mays* (*λ*_max_ = 735 ± 0.7 nm), S. *oleracea* (*λ*_max_ = 736 ± 0.5 nm) and *S. wallisii* (*λ*_max_ = 736 ± 1.4 nm) cannot be significantly distinguished from each other. As far as we know these results show for the first time that PSI complexes from different plant species are significantly different in their spectral properties.

### PSI absorption spectra are similar

**Fig. 3:**
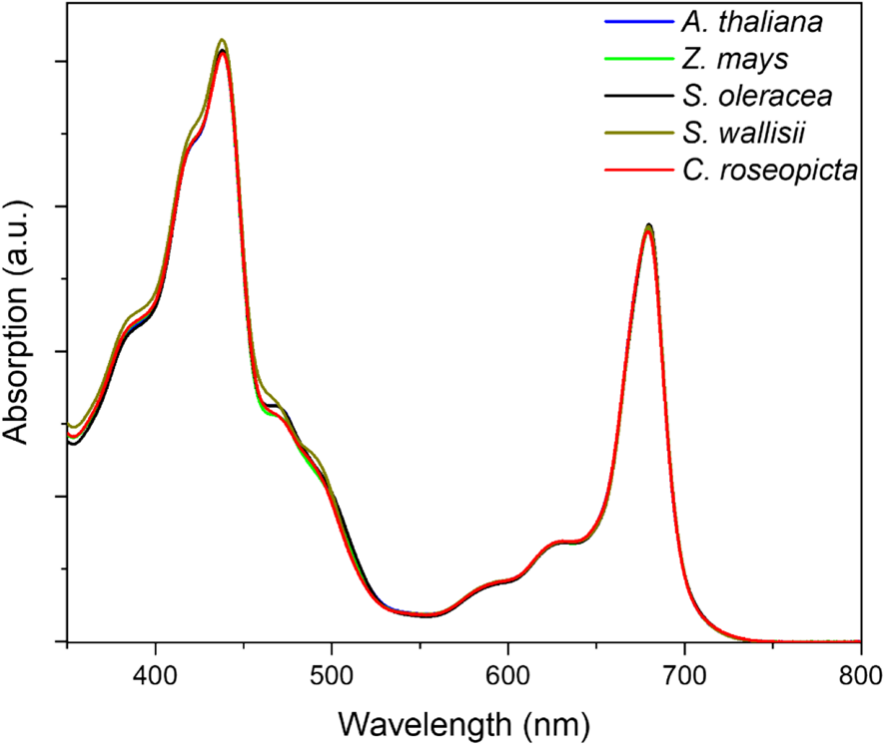
Average absorption spectra of PSI from 5 plant species. Absorption at 750 nm is set to zero and spectra are normalised to the area between 650 and 750 nm. *N* = 4 for Zm, So, Sw and Cr, *N* = 5 for At

Next, absorption spectra of the PSI isolates from the species were recorded, showing minor differences based on four independent sample preparations per plant species (Fig. 3). A small but significant difference is found between *Z. mays* and *S. wallisii* regarding the amount of absorption at 465 nm, resembling Chl *b* absorption (*p* = 0.049 as determined by one-way ANOVA with Tukey test). Although it is possible that PSI from different species have a slightly altered Chl *a*/*b* ratio, contamination with PSII complexes can also explain this deviance. In support for the latter hypothesis, the 77-K spectrum also indicates the PSI samples of *S. wallisii* are more contaminated with uncoupled Chls and/or PSII complexes than the sample of *Z. mays* (Supplementary Fig. S1). When studying the far-red absorption of PSI of the five species, no significant differences are detected. This points out that although PSI fluorescence at 77 K differs significantly between species, the RT absorption spectra are hardly affected by this diversity.

### Variability of RT PSI fluorescence

**Fig. 4:**
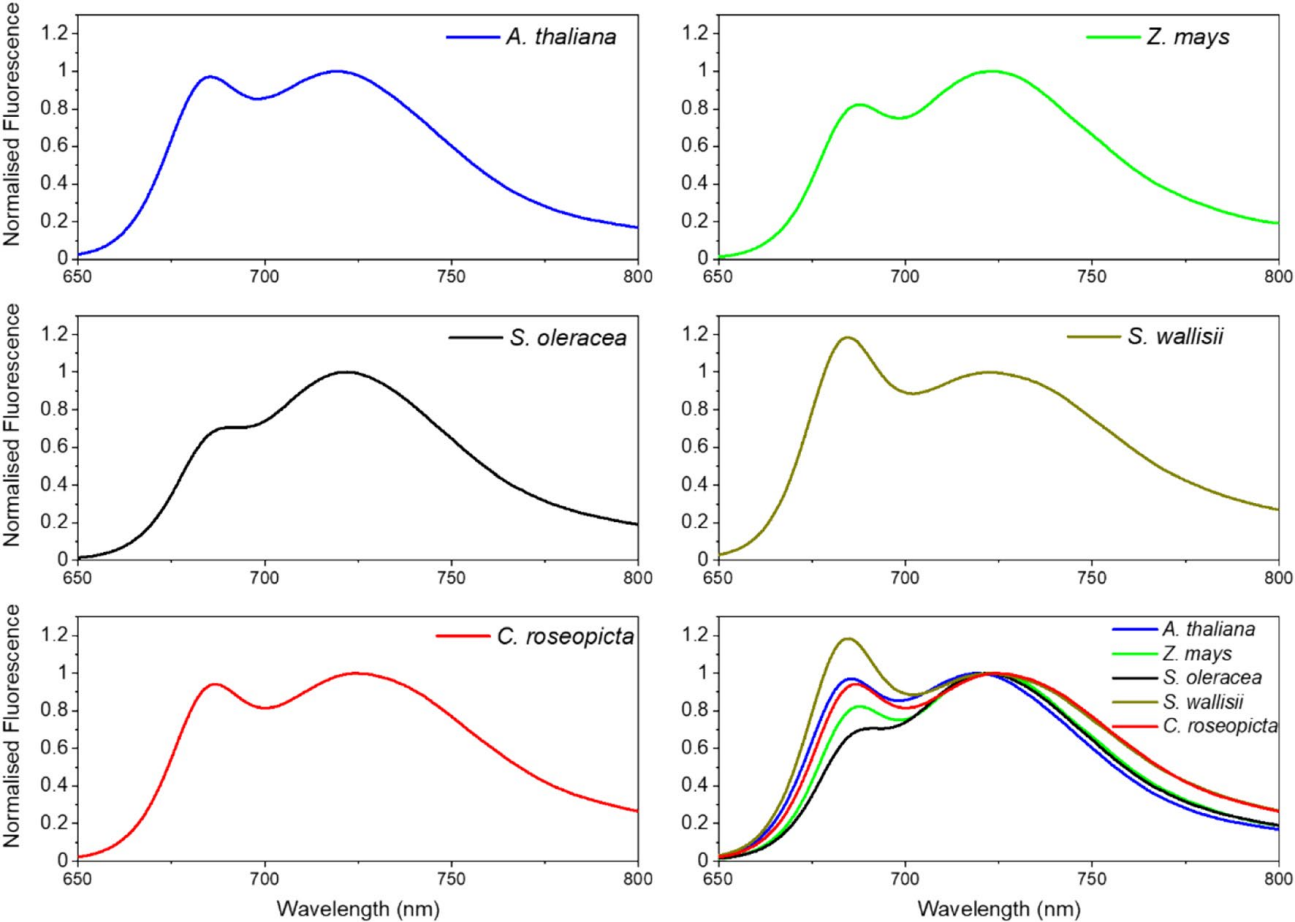
RT steady-state fluorescence spectra of PSI isolates from five plant species at excitation of 435 nm. Spectra are normalised to the maximum fluorescence intensity peak of PSI around 723 nm. All spectra are plotted together in the last panel for comparison

Next, we present the room-temperature steady-state fluorescence spectra that can be used in remote sensing applications as PSI spectrum (Fig. 4). Contrarily to the earlier absorption and 77-K fluorescence spectra, only the spectra with the least contamination are presented, since impurities of PSII or free pigments fluoresce more than PSI and tend to dominate the spectrum. Like the 77-K fluorescence spectra, the RT fluorescence spectra also display variation in the amount of fluorescence in the far-red region. *C. roseopicta* has the most red-shifted fluorescence spectrum and *A. thaliana* the most blue shifted. These differences are apparent both from the values of *λ*_max_, the wavelength at maximum emission, and the red tail of the spectra. All in all this points again towards heterogeneity in PSI of different plant species that was also visible in the 77-K fluorescence spectra.

### Excitation energy trapping by PSI

**Fig. 5:**
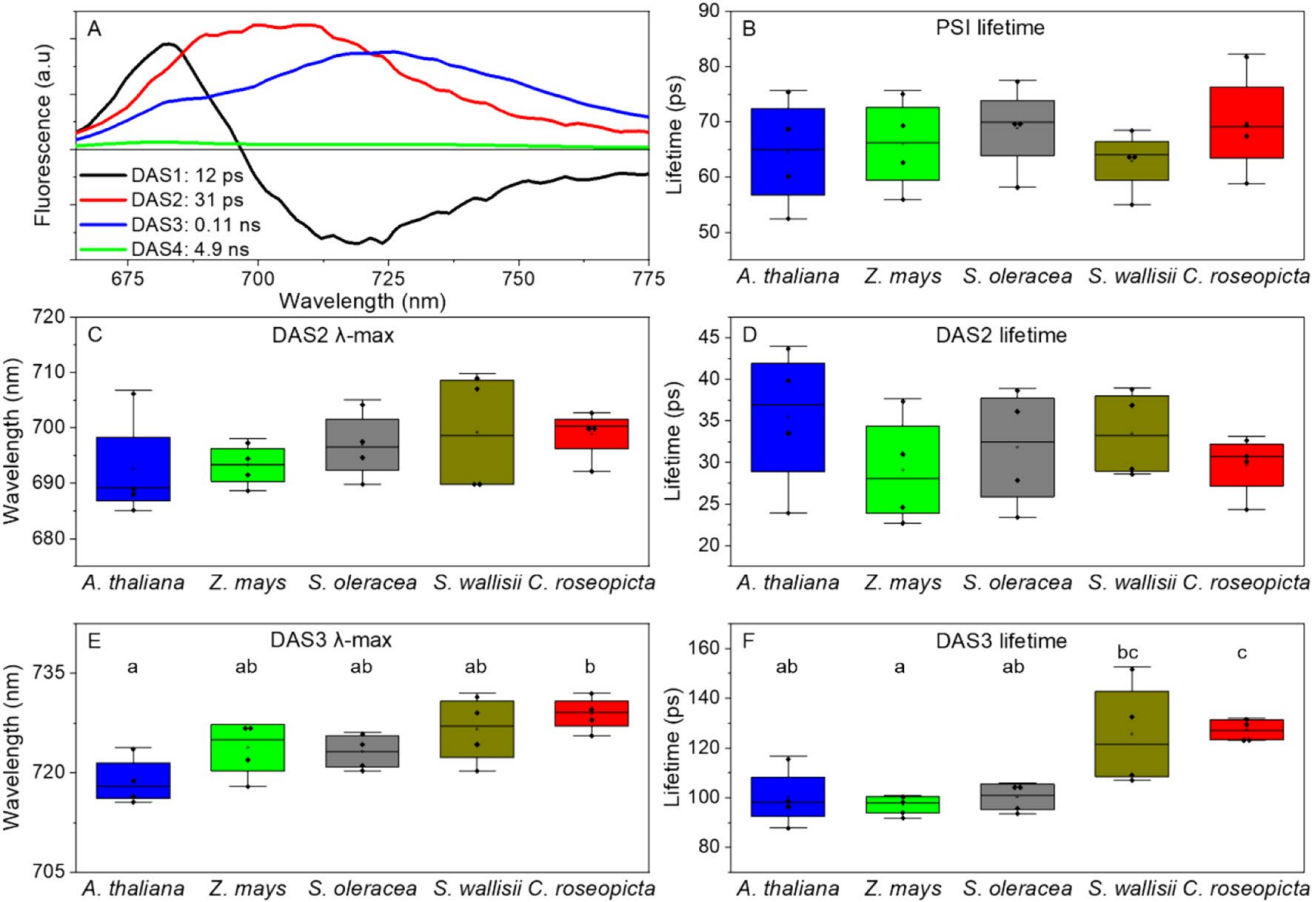
Overview of time-resolved fluorescence spectra, *λ*_max_ and lifetimes. **A** Representative decay-associated spectra of a PSI isolate from *S. oleracea*. **B** Average lifetimes of PSI from five species, calculated from DAS2 and DAS3. **C**, **D** *λ*_max_ and lifetime of DAS2. **E**, **F** *λ*_max_ and lifetime of DAS3. Where applicable, significant groups are indicated with letters, based on a Tukey test (*p* < 0.05). *N* = 4 for all species

Streak camera time-resolved fluorescence measurements allow for accurate determination of both spectra and fluorescence decay kinetics of different components within a sample. In the case of PSI isolates, the data can be fitted with four decay-associated spectra (DAS); 3 DAS for PSI and one for impurities (Fig. 5A). The short-lived DAS1, with lifetimes between 6 and 20 ps, has both positive and negative parts and represents transfer from the bulk Chls to the red-absorbing Chls. DAS 2 and 3, with lifetimes between 23–46 ps and 79–168 ps, respectively, correspond to PSI. DAS2 describes the decay of the bulk Chls, whilst DAS3 is related to red-Chl decay. The lifetime of DAS3 is larger than the one of DAS2 since uphill energy transfer is required for the excitation to reach the reaction centre. DAS4 belongs to impurities of the sample that can either be PSII contamination or free pigments. From the decay-associated spectra of PSI, an average lifetime of the complex can be calculated by taking the sum of the products of the relative area of a DAS and its lifetime for the relevant spectra. Average lifetimes for the different PSIs calculated this way range from 60 to 70 ps at 293 K, which is longer than normally reported for PSI-LHCI (Croce et al. 2000; Ihalainen et al. 2002, 2005; Van Oort et al. 2008; Wientjes et al. 2011a, b; Jennings et al. 2013). The lifetimes of PSI in this study do not differ significantly from each other (Fig. 5B). The same is true for the average lifetime and the *λ*-max of DAS2 (Fig. 5C, D). These results illustrate that the bulk Chls in PSI are conserved between these five species and that their energetic connections to the core complex are comparable. However, significant differences are found in the DAS3 emission maxima and lifetimes (Fig. 5E, F). *A. thaliana* has a blue-shifted *λ*_max_ (719 ± 1.8 nm) and shorter lifetime (100 ± 6 ps) compared to *C. roseopicta* (*λ*_max_ = 729 ± 1.3 nm, lifetime = 127 ± 2 ps)*.* The *C. roseopicta* DAS3 lifetime is also longer than the lifetimes form *Z. mays* (97 ± 2 ps) and *S. oleracea* (100 ± 3 ps). The *S. wallisii* DAS3 lifetime (*λ*_max_ = 727 ± 2.6 nm, lifetime = 126 ± 11 ps) is distinct from *Z. mays.* An increased DAS3 lifetime for a species correlates with a higher *λ*_max_ found in the 77-K fluorescence measurements (Fig. 2).

These combined results illustrate that the fluorescent properties of PSI from different plant species differ. The RT, 77 K and time-resolved fluorescence measurements all demonstrate that the red forms of PSI from *C. roseopicta* are red shifted and the ones from *A. thaliana* are blue shifted compared to the other species. Little differences are found between the red forms of *Z. mays*, *S. oleracea* and *S. wallisii*.

### PSI fluorescence spectrum changes at biologically relevant temperatures

**Fig. 6:**
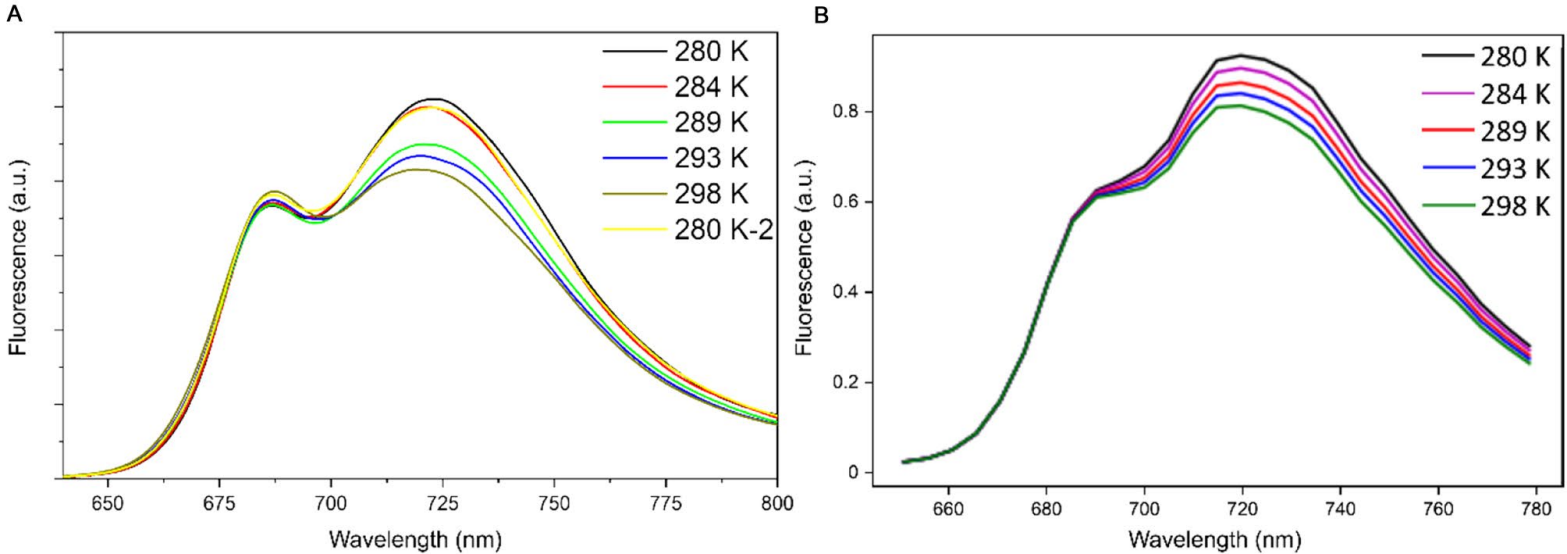
Temperature dependence of steady-state PSI fluorescence spectra with excitation at 435 nm. **A** *S. oleracea* PSI fluorescence spectra recorded at stepwise increased temperatures (280–298 K). Recovery of the original spectrum was checked by cooling back down to 280 K (280 K-2). **B** Steady-state modelled temperature-dependent spectrum of PSI-LHCI. Temperature-dependent transfer rates were calculated via a Boltzmann equilibrium and applied in a model resembling the one from Schiphorst et al. (2022). A more detailed description of the construction of the model can be found in the Supplementary information

Temperature is known to influence the shape and intensity of fluorescence spectra (Croce et al. 1998). Several studies have presented fluorescence spectra recorded at different temperatures in vitro for PSI core, LHCI complexes and PSI (Croce et al. 1998, 2000; Agati et al. 2000). Differences in the amount of fluorescence from the red forms were observed. More specifically, since environmental heat is required to transport excitation energy from the red forms to the PSI core, a decrease in red fluorescence is observed with increasing temperatures. However, the experiments on LHCI were performed at 80–280 K, which makes the spectra less relevant for natural conditions. Fluorescence changes have also been observed in vivo in whole leaves at biologically relevant temperatures, but these changes were attributed to changes in PSII fluorescence (Agati et al. 2000). Here, we present temperature-dependent changes in the PSI fluorescence spectrum from *S. oleracea* between 280 and 298 K (Fig. 6A). A clear decrease in fluorescence from the red forms can be recognised upon increasing the temperature and this fluorescence makes an almost full recovery when cooling back to 280 K. The total area under the graph decreases at higher temperature, indicating faster trapping of excitations by the reaction centre. The peak at ~ 685 nm increases a little upon increasing temperature, but does not drop back when cooling back down to 280 K, most likely pointing towards a minute increase of free pigments. This is in accordance with the slightly incomplete recovery of the red fluorescence peak upon cooling, which points towards a small amount of damage to the PSI complex by either exposure to light or elevated temperatures. Heating to 307 K led to considerably less recovery of the original fluorescence data at 280 K (Supplementary Fig. S2). The area under the fluorescence spectrum from 700 to 800 nm of PSI of *S. oleracea* at 286 K increases to 110% (0.3% SE, *N* = 3 technical replicates) of the area at 293 K and at 298 K; the area is only 93% (0.2% SE, *N* = 4 technical replicates) of the area at 293 K after normalisation to the peak at 685 nm (Fig. 6A). To check if the PSII emission intensity was also affected by temperature the PSII core was isolated and it appeared that PSII was to a substantially larger extent damaged by temperature than PSI (Supplementary Fig. S3). More importantly, the shape of the fluorescence spectrum of PSII is not temperature dependent. This shows that the total in vivo fluorescence does not only vary due to biological reasons, such as quenching of PSII, but also due to abiotic variables, such as a temperature effect on PSI. The temperature-dependent change in PSI fluorescence is comparable to differences found with simple temperature-dependent PSI spectrum modelling (Fig. 6B, Supplementary Fig. S4 and supplemental text), indicating that the differences arising with changing temperature can be explained by Boltzmann equilibrium distribution.

**Fig. 7:**
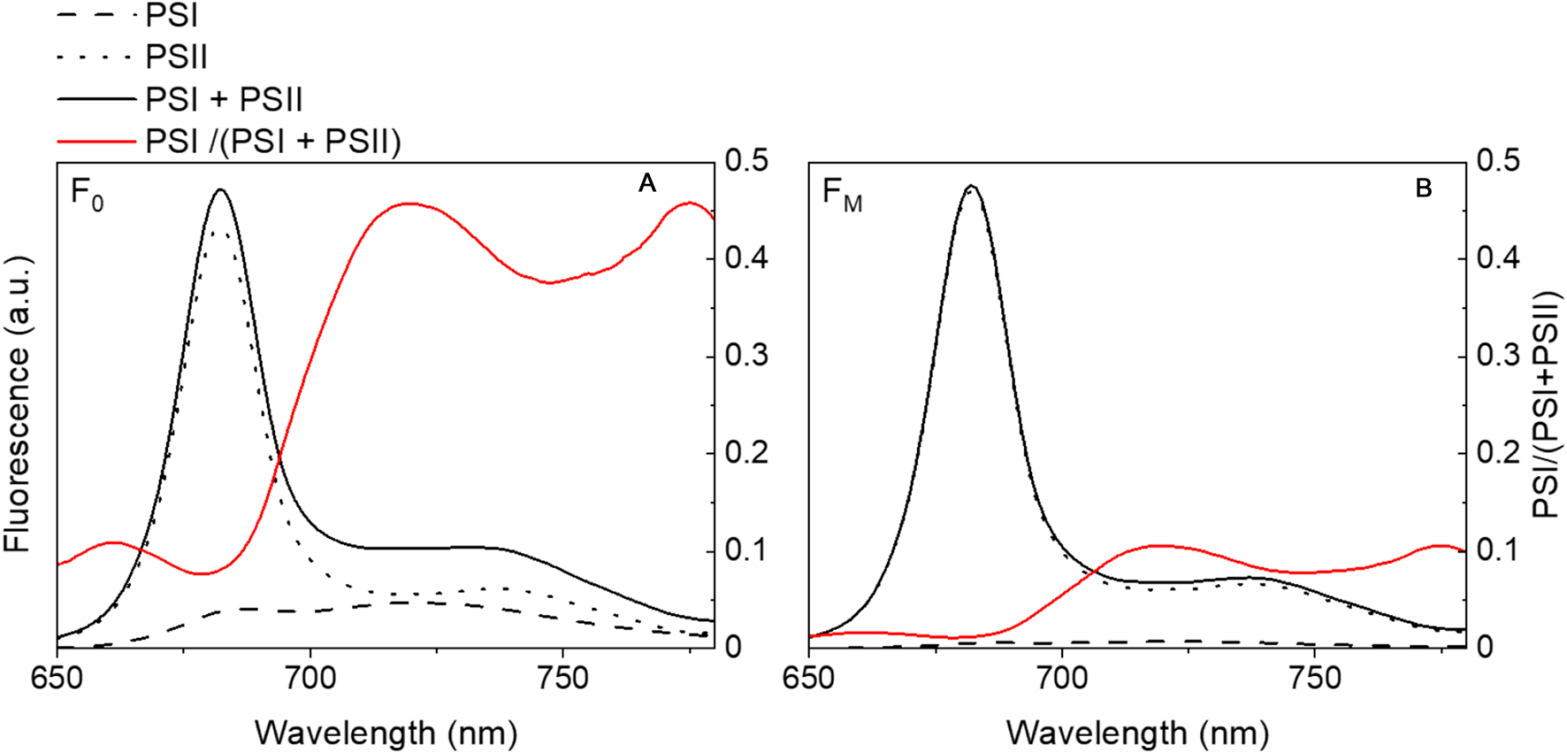
PSI and grana spectra from *S. oleracea* at 293 K. **A** PSI and PSII spectra multiplied with their lifetime considering open PSI and PSII reaction centres (69 and 224 ps, respectively), together with the resulting F_0_ fluorescence spectrum. In red the PSI contribution to the fluorescence. **B** F_M_ situation with closed PSII reaction centres (1.6 ns). A smaller PSI contribution to the total fluorescence is observed in F_M_

Using the PSI and PSII spectra from *S. oleracea*, an estimation of the PSI contribution to the total in vivo chlorophyll fluorescence at different temperatures can be made. The total fluorescence spectrum (the addition of the PSI and PSII spectrum multiplied with their lifetimes) was calculated considering the F_0_ situation in which all PSII reaction centres are open and the F_M_ situation with only closed PSII reaction centres (Fig. 7). Considering the use of an RG9 filter as is common in most modern PAM set-ups (Porcar-Castell et al. 2014; Pfündel 2021), PSI contributes 41% to F_0_ fluorescence. At 280 K this contribution increases to 43% but at 298 K it is 40%. However, this does not change the F_V_/F_M_ ratio (measure for PSII quantum efficiency), which remains 0.78 in these calculations. In F_M_ conditions, the PSI contribution is only 9% and is not significantly influenced by changes in temperature.

## Discussion

All higher organisms depend on oxygenic photosynthesis for their survival. Oxygenic photosynthesis is driven by PSI and PSII. Whilst multiple studies have investigated PSI spectral properties and trapping kinetics (Croce et al. 1996, 1998, 2000; Ihalainen et al. 2002, 2005; Wientjes et al. 2011a, b; Jennings et al. 2013; Bos et al. 2017; Molotokaite et al. 2017; Chukhutsina et al. 2020), little is known about how these properties differ between plant species. Here we isolated PSI-LHCI from five different plant species to compare their protein composition and optical properties.

We found significant differences in the red forms of the various PSI complexes, based on 77 K steady-state fluorescence measurements. These differences most likely arise from differences in Lhca3 and Lhca4, the LHCI subunits in which the red Chls are located (Morosinotto et al. 2003; Wientjes and Croce 2011). We hypothesised that plants adapted to life in the canopy shade (e.g. *S. wallisii* and *C. roseopicta*) are likely to have evolved more red-shifted Chls than plants adapted to full-sun conditions, such as crop plants (*Z. mays* and *S. oleracea*) or pioneer species (*A. thaliana*). The 77 K fluorescence spectrum of *C. roseopicta* is indeed found to be red shifted and the one of *A. thaliana* blue shifted with regard to the other species. The lifetime of the DAS associated with the red forms and the RT fluorescence spectra is in agreement with this result. However, the absorption spectra of the complexes of the different species do not differ in the part > 700 nm, which indicates that red-shifted emission is not correlated with a detectable change in absorption of far-red photons. Our hypothesis that PSI of species that are adapted to shadow-rich conditions can use more far-red light for photosynthesis is not confirmed for the plants investigated in this study.

Since *A. thaliana* is a model species, its PSI 77 K fluorescence spectra have been reported several times (Drop et al. 2011; Wientjes et al. 2011a, b; Galka et al. 2012; Benson et al. 2015; Chukhutsina et al. 2020), with an emission maximum ranging between 730 and 733 nm. Our determined emission maximum of 731 ± 0.4 nm is in agreement with these studies. Also the emission maximum of *S. oleracea* of 736 ± 0.5 nm is similar to previously determined values (Pålsson et al. 1995). However, Chukhutsina et al. found for *Z. mays* an emission maximum of 730 nm (Chukhutsina et al. 2020), which does not fall within our determined range (*λ*_max_ = 735 ± 0.7 nm). The differences between these studies are surprising considering the determined emission maxima of *A. thaliana* in these studies are similar. Moreover, only small variations have been found between studies that recorded the 77 K emission maximum of the same species, a range of 730–733 nm for A. thaliana in 5 studies and a range of 733–736 nm for P*. sativum* based on three studies (Akhtar and Lambrev 2020; Wang et al. 2021; Yan et al. 2021), indicating that differences in isolation procedure do not lead to large deviations. It has also been shown that various light conditions during growth do not lead to a shift in the PSI fluorescence maximum in *A. thaliana* (Wientjes et al. 2013a, b). In our opinion, differences between cultivars of *Z. mays* are the most likely explanation for the deviation between the studies, which makes *Z. mays* an interesting species to study further.

In this study, we found that *C. roseopicta* has the most red-shifted *λ*_max_ reported at 77 K for flowering plants (*λ*_max_ = 740 ± 0.8 nm for the isolated complex and *λ*_max_ = 745 nm in thylakoids). The combined data on PSI from different angiosperms suggest that emission maxima of flowering plants fall in a range of about 15 nm between 730 and 745 nm at 77 K. More diversity is found between the red forms of other species of oxygenic photosynthetic organisms (Chen et al. 2020; Huang et al. 2021; Yan et al. 2021). The moss *P. patens* with a similar LHCI build-up as angiosperms is found to have an emission maximum at 727 nm at 77 K (Yan et al. 2021). The blue shift of *λ*_max_ in comparison to flowering plants is due to the replacement of Lhca4 with Lhca5, which lacks red-shifted Chls (Wientjes et al. 2009; Yan et al. 2021). As such, the LHCI of PSI of *P. patens* is composed of an Lhca1/5 and an Lhca2/3 dimer. The red forms of Lhca3, in the Lhca2/3 dimer, are responsible for the 727-nm emission (Wientjes et al. 2011a, b). The 77-K emission of the PSI complex of *C. reinhardtii* is about 18 nm blue shifted compared to that of *A. thaliana* (Drop et al. 2011; Le Quiniou et al. 2015; Huang et al. 2021). Interestingly, their trapping times are similar, mainly because LHCI of *C. reinhardtii* is larger than the one of *A. thaliana*, which increases the lifetime to a similar value (Le Quiniou et al. 2015). Within algae, variation in antennae size and red shift of red forms is identified, but no species with 77-K emission maxima as red shifted as those of angiosperms have been reported (Swingley et al. 2010; Perez-Boerema et al. 2020). Cyanobacteria lack LHCI, but in several species (more red shifted) red forms that arise from oligomerisation of PSI have been detected, even up to *λ*_max_ of fluorescence of 760 nm at 6 K in *Spirulina platensis* (Karapetyan et al. 1999, 2014; Gobets et al. 2001).

In PAM measurements on whole leaves, the PSI contribution to the total fluorescence is usually neglected (Pfündel et al. 2013; Giovagnetti et al. 2015). However, several studies have shown that the PSI contribution can reach up to 40% to the minimal fluorescence level (F_0_) and up to 10% to the maximum fluorescence value (F_M_) at detection wavelengths of 720 nm or larger (Agati et al. 2000; Franck et al. 2002). It has been determined that PSI can contribute up to 25% in *A. thaliana*, 50% in *Z. mays* (Pfündel et al. 2013), and 45% in *Prunus laurocerasus* L. (Pfündel 2021) to the fluorescence at detection wavelengths > 700 nm. Our calculations of the PSI contribution to F_0_, based on the PSI and PSII spectra of *S. oleracea* at 293 K, correspond to these values. We also show that changes in temperature between 280 and 298 K do not significantly alter this contribution, nor does it change the F_V_/F_M_ ratio. Therefore, PSI correction of PAM data is essential for a correct interpretation of the PSII quantum yield, but the correction does not have to be carried out with a temperature-dependent PSI spectrum. However, variations in PSI contribution in F_0_ and resulting changes in F_v_/F_M_ can arise between different plant species.

Put together, our results show variability of the PSI fluorescence spectrum between different angiosperms. More specifically, the shadow-tolerant plant *C. roseopicta* has the most red-shifted emission maximum for PSI reported for flowering plants. However, its absorption spectra does not differ significantly from our other studied plants in the far-red region which challenges the view that red-shifted red forms allow plants to use a broader light spectrum. In addition, we showed that biologically relevant temperature changes have a substantial effect on the emission spectrum of PSI.

## Supplementary Information

Below is the link to the electronic supplementary material. The data presented in this study is publicly available at the 4TU Centre for Research Data (4TU.ResearchData) via 10.4121/20029532.

Supplementary file1 (DOCX 1982 KB)
