# Supplementary material for: Spectral diversity of photosystem I from flowering plants
Source: Photosynth Res. 2022 Oct 19;155(1):35–47. doi: 10.1007/s11120-022-00971-2 (PMC9792416; doi:10.1007/s11120-022-00971-2)
Supplement: Supplementary file 1 — Supplementary file1 (DOCX 1982 KB) [file 11120_2022_971_MOESM1_ESM.docx]

# Supplementary Information

Spectral Diversity of Photosystem I from Flowering Plants

**Photosynthesis Research**

Peter R. Bos^1^, Christo Schiphorst^1^, Ian Kercher^1^, Sieka Buis^1^, Djanick de Jong^1^, Igor Vunderink^1^ and Emilie Wientjes^1^.

^1^Laboratory of Biophysics, Wageningen University, P.O. Box 8128, 6700 ET Wageningen, The Netherlands

Corresponding author: Emilie Wientjes, [emilie.wientjes@wur.nl](mailto:emilie.wientjes@wur.nl) ORCID: 0000-0003-2625-8045


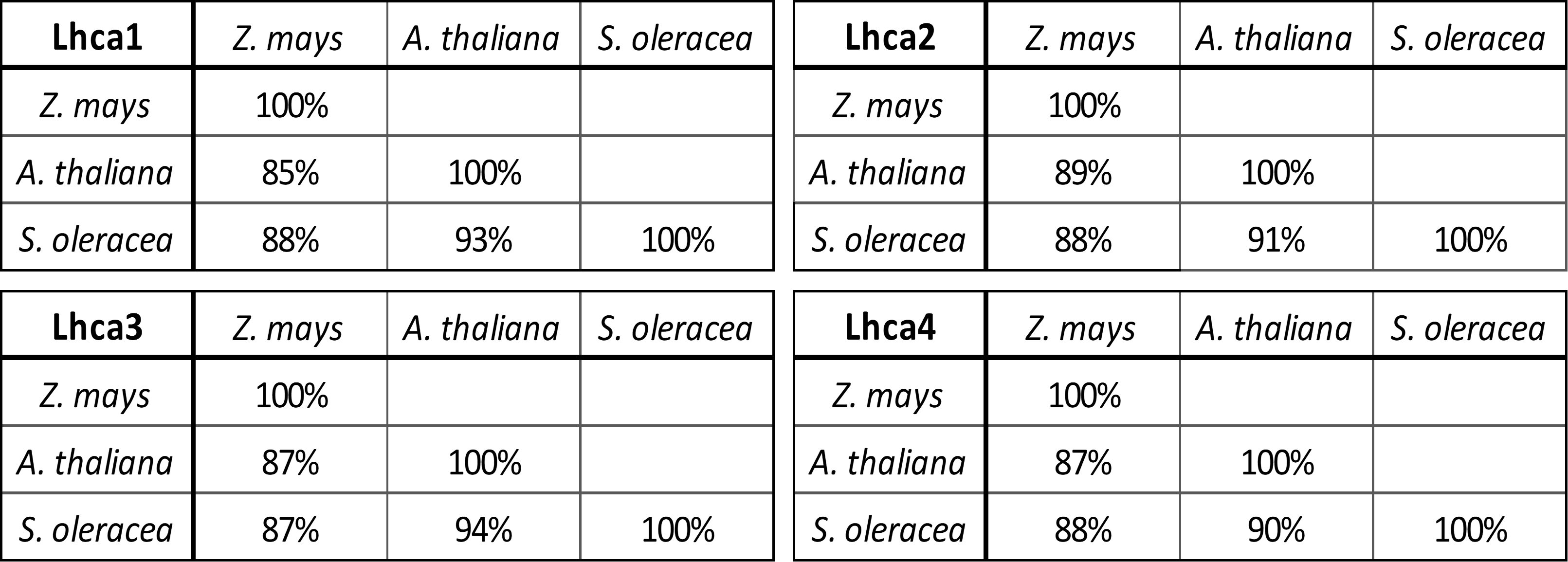


Supplemental Table S1 Percent identity of the protein sequences without chloroplast transit peptide of Lhca1-4 from S. oleracea, Z. mays and A. thaliana. Percent identity was determined by multiple sequence alignment using Clustal Omega (Sievers, Wilm et al. 2011, McWilliam, Li et al. 2013).


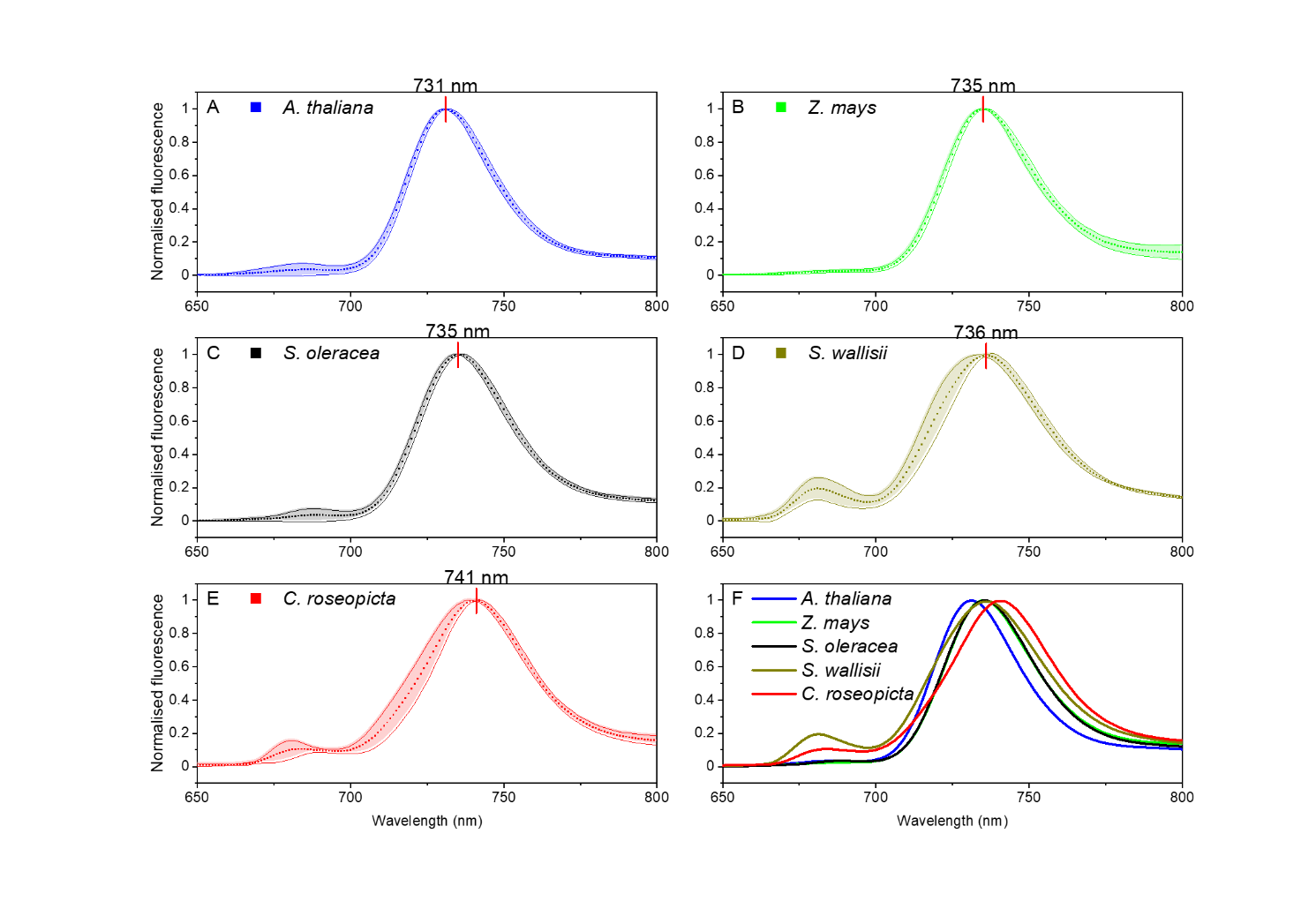


Supplemental figure S1 77 K fluorescence spectra of five species with N=4 of all independent replicas on different PSI-LHCI preparations. The wavelength of emission maximum is given for all spectra. Average spectra are plotted together in figure F to appreciate the differences.


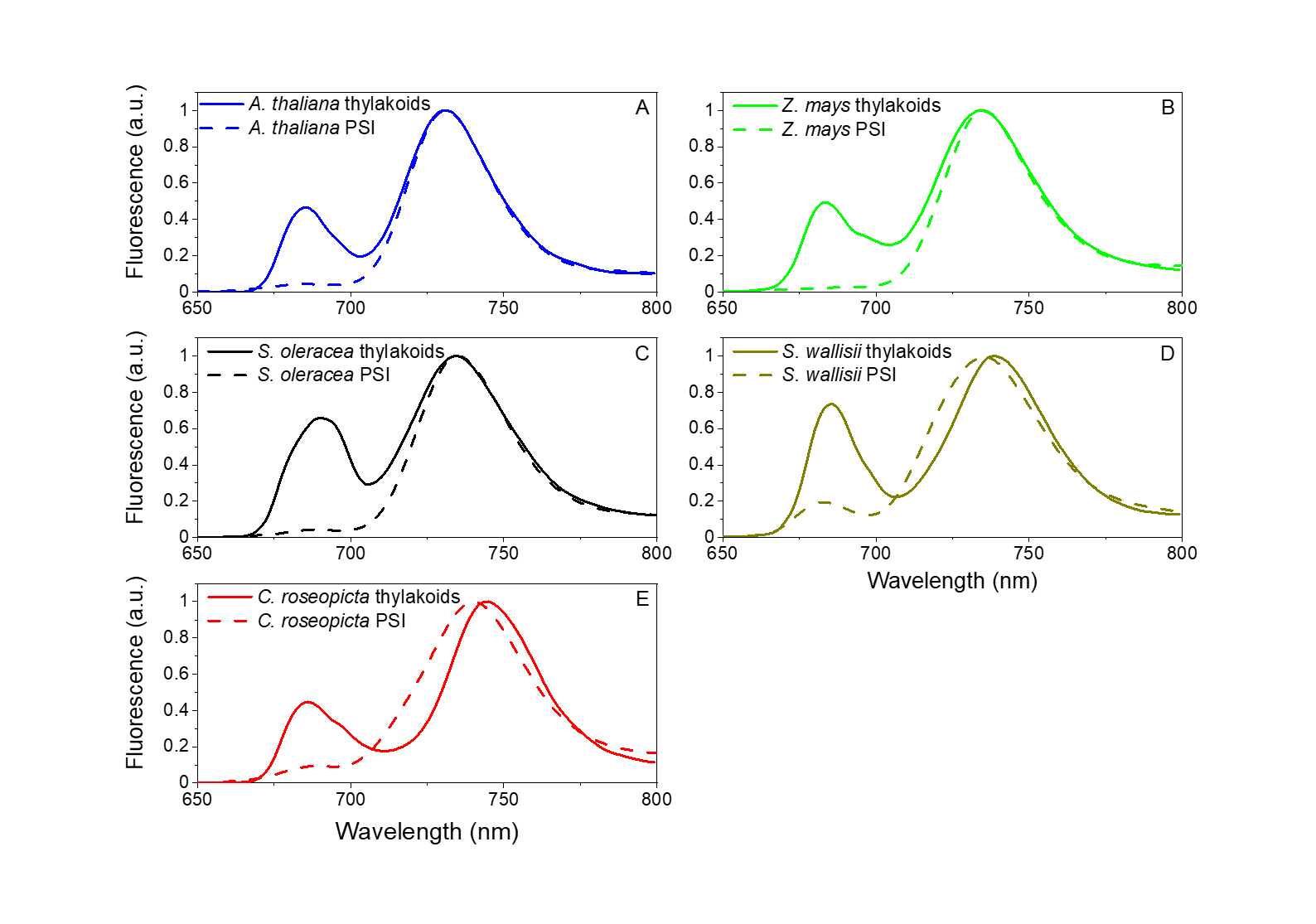


Supplemental figure S2 77 K fluorescence spectra of isolated thylakoids (solid lines) and isolated PSI (dashed lines) from five plant species. Single spectra were recorded for thylakoids. An average from 4 spectra was taken for the PSI spectra.


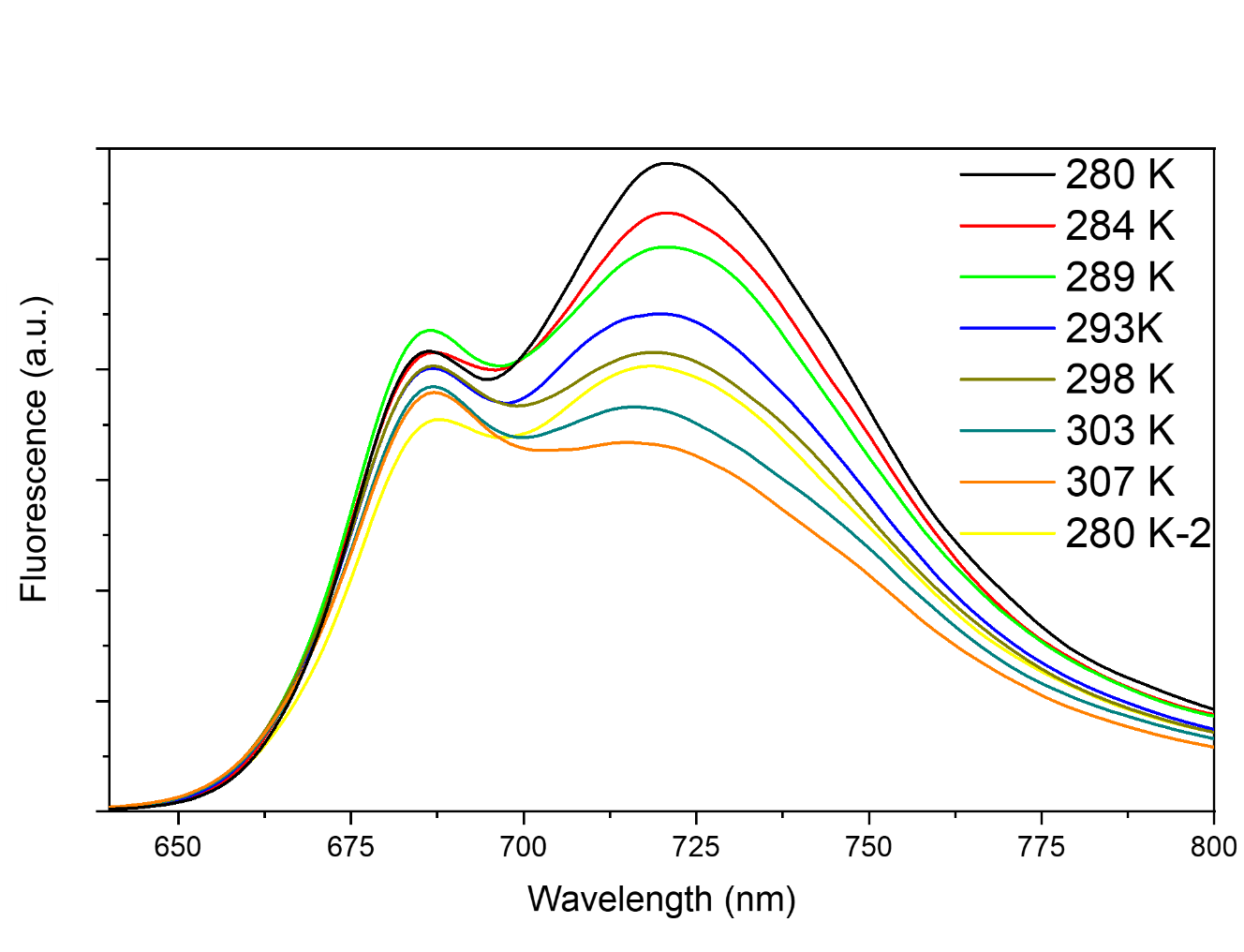


Supplemental figure S3 Temperature dependence of the RT steady-state PSI-LHCI fluorescence spectrum with excitation at 435 nm. S. oleracea PSI-LHCI fluorescence spectra recorded at stepwise increased temperatures (280-307 K). Recovery of the original spectrum was regarded by cooling back down to 280 K (280 K-2). The incomplete recovery of the fluorescence peaks at ~685 nm and ~720 nm points towards damage of the PSI-LHCI complex.

## Supplemental text


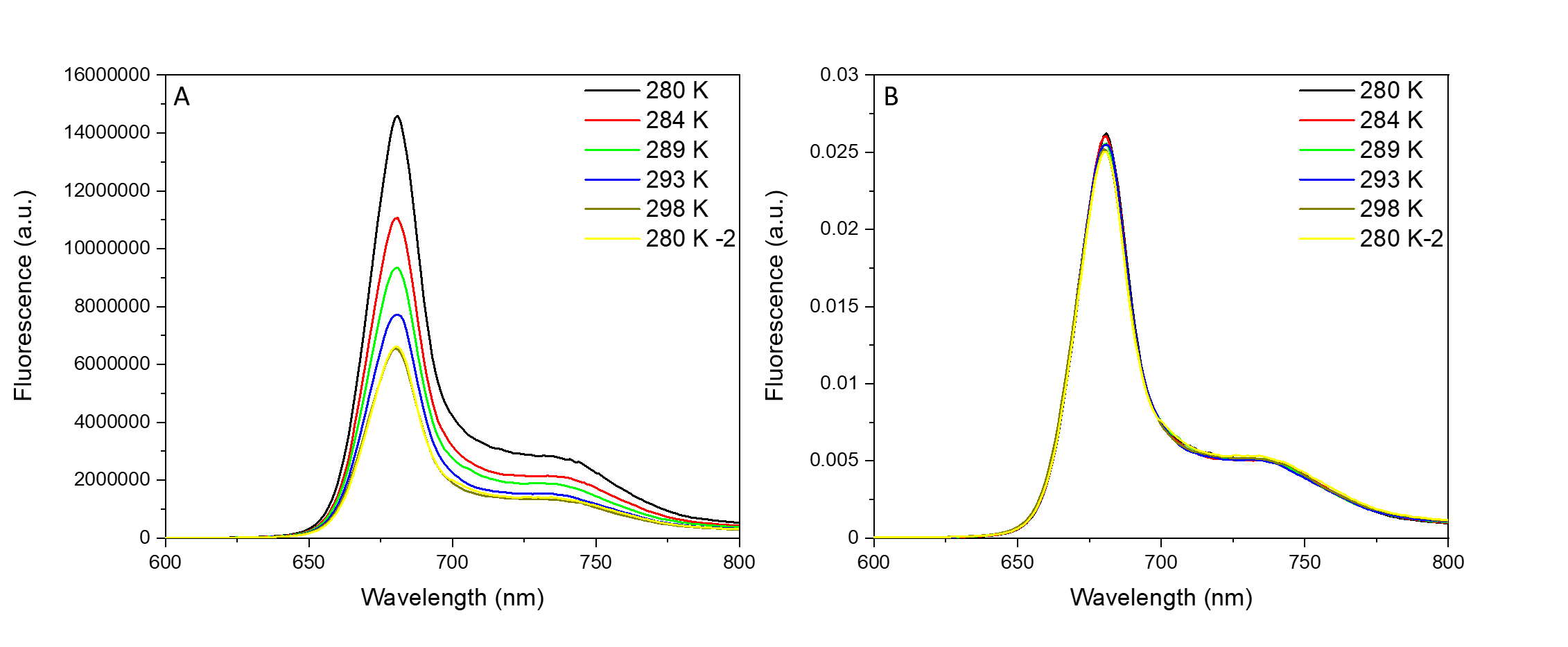


Supplemental figure S4 PSII temperature dependence. A: Unmodified spectra of isolated PSII at increasing temperatures. The second spectrum recorded at 280 K is taken to regard damage to the photosystem due to heat or light. Fluorescence intensity drops after every measurement at an increasing temperature, showing damage to the photosystem. No drop intensity when cooling from 298 K to 280 K indicates that temperature and not excess light is the cause for damage to PSII. B: Spectra from A normalised to total area. Little change in the shape of the spectra is observed in response to temperatures changes.

A minimal modelling system was used to simulate the changes in the steady-state fluorescence spectrum of PSI-LHCI at different biologically relevant temperatures. Two pigment pools were used for the simulation, PSI-LHCI and the red forms (supplementary figure S4A). Since the ratio of downhill and uphill transfer is dominated by Boltzmann statistics, transfer rates between the pigment pools was calculated based on Boltzmann statistics $k_{j\to i}=k_{i\to j}*\frac{n_{i}}{n_{j}}*e^{\frac{-(\varepsilon_{i}-\varepsilon_{j})}{k_{B}*T}}$ where the energy levels (ε*_x_*) are given by $\varepsilon_{x}=\frac{h*c}{\lambda_{x}}$. In which $\lambda_{i}=690nm$ and $n_{i}=139 Chls$ were used for PSI-LHCI and $\lambda_{j}=723nm$ and $n_{j}=4 Chls$ for the red forms, *h* is Planck’s constant, *c* is the speed of light, $k_{B}$ is the Boltzmann constant and *T* is the temperature in K. The transfer rate from PSI-LHCI ($k_{i\to j}$) was kept constant and the reverse transfer rate from the red forms to PSI-LHCI ($k_{j\to i}$) therefore only depended on the temperature and increased with increasing temperatures. The transfer and decay rates were used to model the decay kinetics of the individual pigment pools at the different temperatures, see supplementary figure S4B. Emission spectra of PSI-LHCI and the red forms were taken from Schiphorst et al. (Schiphorst, Achterberg et al. 2022) (supplementary figure S4C), and multiplied with the decay kinetics to give a time-resolved fluorescence spectrum (supplementary figure S4D). Integrating the time-resolved fluorescence spectrum over the time-axis provides the steady-state fluorescence spectrum (supplementary figure S4E).

| 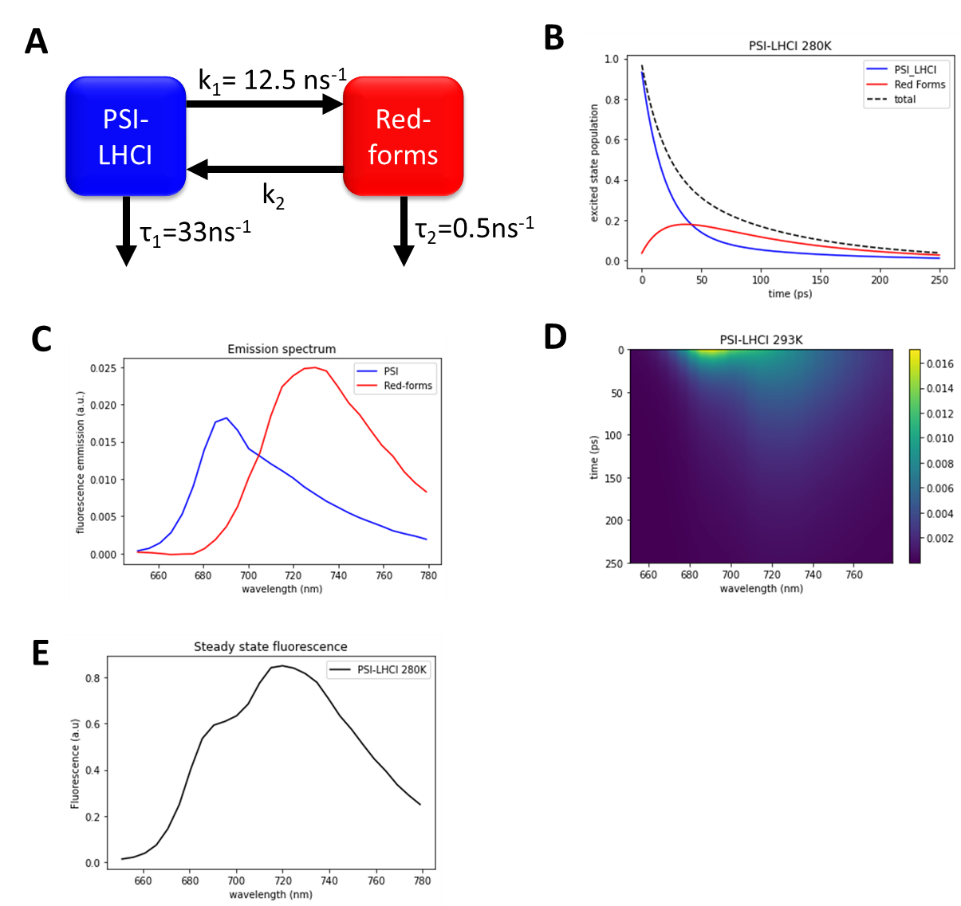 |
| --- |

Supplemental figure S5 Kinetic modelling approach. A) Minimal kinetic scheme for the PSI-LHCI and red form energy distribution. B) The population change over time of bulk Chl (PSI-LHCI) and red forms. C) The fluorescence emission spectra of PSI and the red forms. D) A 3D intensity map of PSI-LHCI over time and emission wavelength. E) Steady state fluorescence spectrum of PSI-LHCI calculated from the time-resolved 3D intensity map.
